# UbiD domain dynamics underpins aromatic decarboxylation

**DOI:** 10.1038/s41467-021-25278-z

**Published:** 2021-08-20

**Authors:** Stephen A. Marshall, Karl A. P. Payne, Karl Fisher, Gabriel R. Titchiner, Colin Levy, Sam Hay, David Leys

**Affiliations:** 1 Manchester Institute of Biotechnology, University of Manchester, Manchester, UK; 2 Present Address: Chemistry Research Laboratory, University of Oxford, Oxford, UK

**Keywords:** X-ray crystallography, Enzyme mechanisms, Enzymes

## Abstract

The widespread UbiD enzyme family utilises the prFMN cofactor to achieve reversible decarboxylation of acrylic and (hetero)aromatic compounds. The reaction with acrylic compounds based on reversible 1,3-dipolar cycloaddition between substrate and prFMN occurs within the confines of the active site. In contrast, during aromatic acid decarboxylation, substantial rearrangement of the substrate aromatic moiety associated with covalent catalysis presents a molecular dynamic challenge. Here we determine the crystal structures of the multi-subunit vanillic acid decarboxylase VdcCD. We demonstrate that the small VdcD subunit acts as an allosteric activator of the UbiD-like VdcC. Comparison of distinct VdcCD structures reveals domain motion of the prFMN-binding domain directly affects active site architecture. Docking of substrate and prFMN-adduct species reveals active site reorganisation coupled to domain motion supports rearrangement of the substrate aromatic moiety. Together with kinetic solvent viscosity effects, this establishes prFMN covalent catalysis of aromatic (de)carboxylation is afforded by UbiD dynamics.

## Abstract (web summary)

Understanding the structure and dynamics of enzymes is important for a number of applications. Here, the authors report on the crystal structure of vanillic acid decarboxylase, and show how the dynamics of the UbiD superfamily enzymes relate to the covalent catalysis of aromatic (de)carboxylation.

## Introduction

The processing and utilisation of abundant (hetero)aromatic compounds derived from biomass is a sustainable alternative that reduces reliance on oil derived compounds^1–4^. Recent studies have demonstrated that UbiD enzymes can be used for (hetero)aromatic C–H activation at ambient conditions, providing a route to corresponding acids and derivative compounds^5–7^. Studies have also shown the potential of using UbiD enzymes in cascades in the production of *cis,cis*–muconic acid^8,9^ and in 1,3-butadiene production^10^; both valuable chemicals in the production of synthetic polymers.

The UbiD enzyme family is ubiquitous in microbes and is frequently associated with the flavin prenyltransferase, UbiX. The latter produces the prFMN cofactor required for the UbiD-mediated reversible decarboxylation of unsaturated acids (Supplementary Fig. 1)^11^. The UbiD enzyme family can operate on a wide range of unsaturated acrylic, aromatic and heteroaromatic acids, although individual UbiD enzyme substrate specificity has proven to be relatively narrow^12^. Whilst most enzymes function as decarboxylases under physiological conditions, some anaerobic organisms initiate degradation of recalcitrant aromatic compounds such as benzene or naphthalene via UbiD mediated carboxylation^13–15^.

**Fig. 1:**
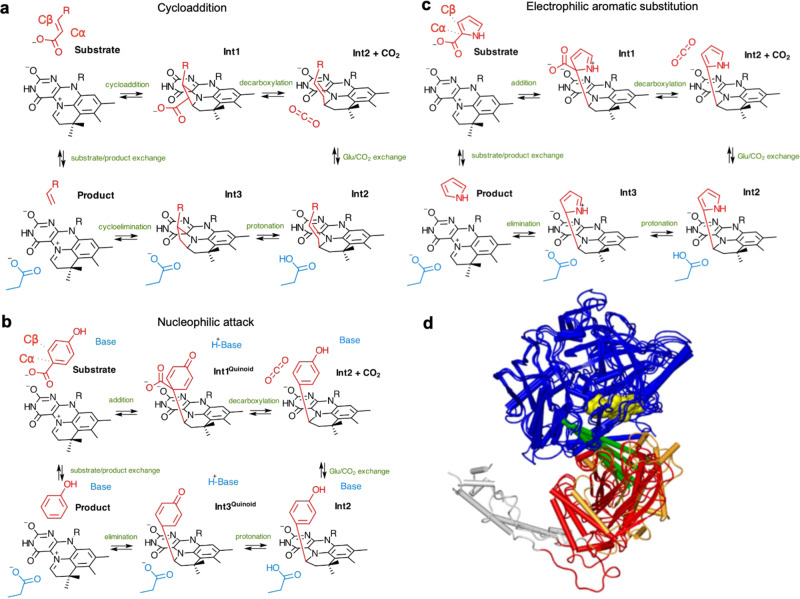
Mechanism and structures of the UbiD enzyme family. **a** Proposed 1,3 dipolar cycloaddition Fdc1 mechanism^16,17^. **b** A substrate nucleophilic attack mechanism proposed for phenolic substrates^18^. **c** Electrophilic aromatic substitution mechanism postulated for PA0254-mediated decarboxylation of pyrrole-2-carboxylate^19^. All mechanisms share a common **Int2** intermediate with an *sp*^2^ hybridized substrate Cα. Transient dearomatisation occurring for **Int1** and **Int3** species presents a significant energy barrier in each case. prFMN-R = phosphoribityl moiety (omitted for clarity) **d** Overlay of UbiD monomers anchored on the prFMN binding domain (in blue). The oligomerisation domains of Fdc and PA0254 are shown in orange (representing a closed conformation), while UbiD, HmfF and AroY are shown in open states (in red). The C-terminal helix is shown in grey. The hinge region is located within a helix (in green) connecting the oligomerisation and prFMN binding domains, close to bound cofactor (in yellow spheres).

A range of studies with the model system fungal Fdc1 (ferulic acid decarboxylase) have established that a reversible 1,3-dipolar cycloaddition between the acrylic acid dipolarophile and the prFMN^iminium^ azomethine ylide occurs via three distinct intermediates^16,17^ (Fig. 1a). Recent protein engineering studies have established Fdc1 can be evolved to accept carboxylated (hetero)aromatic compounds (i.e. where Cα contributes to the (hetero)aromatic ring), although catalytic rates remain low^5^. Covalent catalysis with (hetero)aromatic substrates requires transient dearomatisation of the substrate. A range of mechanisms have been proposed for those UbiD enzymes that catalyse decarboxylation of phenolic (Fig. 1b)^18^ and heteroaromatic acids (Fig. 1c)^6,19^. These all propose formation of a series of covalent adducts between the prFMN C1’ and the substrate Cα (*i.e*. **Int1, 2** and **3**). In the case of the Fdc1 mediated reaction with acrylic acids, the structure of various intermediates reveals considerable strain is imposed by the active site, guiding the reaction along^17^. However, in the case of aromatic substrates, it remains unclear how the enzyme active site can accommodate the substantial rearrangement of the aromatic moiety that accompanies the transition between various prFMN-adduct intermediates. Furthermore, the inherent stability of the **Int2** species afforded by re-aromatisation of the substrate moiety (as compared to **Int1** and **3**) presents an inherent obstacle to rapid turnover.

It has been suggested that UbiD aromatic acid decarboxylases could accommodate the postulated substrate rearrangement by virtue of domain motion dynamics^18,20^. Indeed, the active site sits at the interface of the prFMN binding and oligomerisation domains, and distinct UbiD enzyme structures fall into either open or closed conformational states (Fig. 1d). However, only the PA0254 (also known as HudA) enzyme has previously been shown to adopt both conformations with the transition between both conformations apparently linked to cofactor binding^19^. Hence, only the dimeric and oxygen-tolerant Fdc1 and PA0254 are representatives of the proposed closed state and are accompanied by substrate or ligand complex structures. In contrast, the hexameric and oxygen-sensitive (hetero)aromatic (de)carboxylases such as HmfF, AroY, and UbiD for which crystal structures are available all adopt open conformations and have yet to yield detailed insights into substrate binding^6,18,20^; the substrates of these enzymes are detailed in Supplementary Table 1.

Furthermore, aside from the obligate requirement for UbiX shared by all UbiDs, a number of the aromatic acid (de)carboxylases are reported to require additional proteins for activity. The vanillic acid decarboxylases for example require a small (<~10 kDa) accessory protein VdcD for activity, in addition to the UbiD homolog VdcC^21^. While some UbiD accessory proteins have annotated functions (such as the phosphatase and kinase associated with the phenol phosphate carboxylase enzyme^22^), the role of many of the smaller proteins remains unknown. Interestingly, there is no sequence similarity between VdcD, the phenol phosphate carboxylase small subunit (PpcGamma), or LpdD involved in gallic acid decarboxylation in *Lactobacillus plantarum*^23^.

Here we detail crystal structures of the *Sedimentibacter hydroxybenzoicus* VdcCD enzyme and demonstrate that VdcC forms a tight complex with VdcD, a zinc ribbon protein that allosterically regulates VdcC activity. A comparison of distinct VdcCD crystal structures reveal the presence of open and closed states and establishes that VdcC can undergo a large scale prFMN-binding domain motion, dramatically affecting active site volume. Combined with docking studies and kinetic solvent viscosity effects, our data reveals the structural reorganisation coupled to domain motion supports the rearrangement of the substrate aromatic moiety. This establishes prFMN covalent catalysis of aromatic (de)carboxylation is afforded by UbiD dynamics.

## Results

### A VdcCD complex is required for activity

**Fig. 2:**
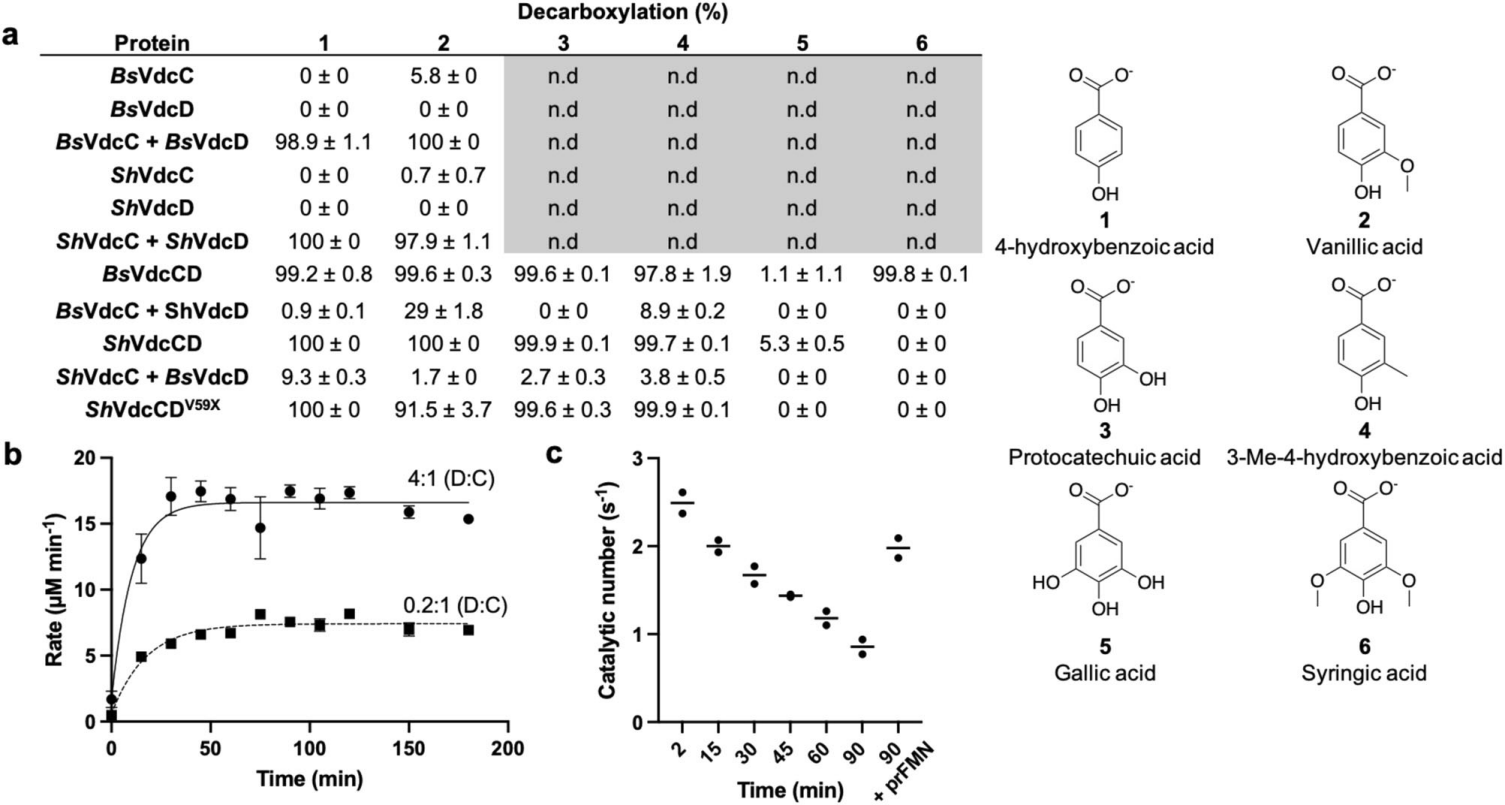
Activity of Vdc proteins. **a** Activity of reconstituted Vdc proteins. Significant activity is only seen when both VdcC and VdcD are present. Protein obtained through co-purification is denoted as VdcCD. Where proteins were purified separately and combined in vitro, the label VdcC + VdcD is used. Substrate specificity of VdcCD enzymes from *Bacillus subtilis* and *Sedimentibacter hydroxybenziocus* show similar substrate ranges, with the exception of syringic acid. Non-cognate VdcD can be used for the limited activation of VdcC. Deletion of the proline rich tail of VdcD has little effect on the activity of VdcCD. Data shown as mean ± SEM *n* = 2 independent replicates. **b** Effect of VdcC:VdcD ratio on decarboxylation rates of 100 µM PHB. A sub-equimolar ratio results in slower decarboxylation of PHB, suggesting a non-transient interaction between VdcC and VdcD. Data are represented by mean ± SEM, *n* = 3 technical replicates. Data trends seen in three independent replicates. **c** Decrease in activity of *Sh*VdcCD with 100 µM PHB in aerobic conditions. Addition of prFMNH_2_ partially restores activity, all data points shown, bar represents mean. Data from two independent replicates.

His-tagged versions of the VdcC proteins from *Bacillus subtilis* (*Bs*VdcC) and *Sedimentibacter hydroxybenzoicus* (*Sh*VdcC; formerly *Clostridium hydroxybenzoicum*^24^) were co-expressed with *Pseudomonas aeruginosa* UbiX in *Escherichia coli* (BL21 DE3). However, following purification, the UV-vis spectrum of the isolated preparations did not provide any evidence of cofactor binding. We tested the decarboxylation activity of both VdcC enzymes reconstituted in vitro with prFMN using vanillic acid and 4-hydroxybenzoic acid (PHB) as substrates. Little to no activity was detected for either VdcC (Fig. 2a), in line with previous reports that VdcD is essential for activity^25,26^. The corresponding His-tagged VdcD subunits were therefore expressed in *E. coli*, and purified VcdD was added to VdcC preparations prior to in vitro prFMN reconstitution. When assayed, the VdcC + VdcD reconstituted samples demonstrated considerable activity in contrast to isolated VdcC. This demonstrates a strict requirement of both VdcC and VdcD for activity. We tested the effect of VdcC:VdcD stoichiometry on activity levels of the *Sedimentibacter hydroxybenzoicus* enzyme. It was found that, while keeping the concentration of VdcC constant, a sub-stoichiometric 0.2:1 D:C ratio led to substantially lower activity when compared with excess D (4:1 D:C ratio) conditions (Fig. 2b). This suggests that VdcD is likely to form an integral part of the catalytic species, as opposed to a transient role in the VdcC-prFMN complex maturation, for example. To confirm that a VdcCD complex is formed, we respectively co-expressed both *Bs*VdcC/D and *Sh*VdcC/D enzymes, whereby only one of the subunits was tagged with a hexa-histidine tag. We found that tagging of either VdcC or D subunit resulted in co-purification of both proteins, yielding the VdcCD complex in both cases. Unfortunately, despite co-expression of UbiX, this procedure did not yield the *holo*-VdcCD without in vitro reconstitution.

### The VdcCD prFMN cofactor is inactivated by oxygen

To confirm previous reports of VdcCD oxygen sensitivity, we reconstituted *Sh*VdcCD under anaerobic conditions with prFMNH_2_. The *holo*-VdcCD was exposed to aerobic conditions and initial decarboxylation activity with PHB was measured at multiple time points over a 90 min period. Activity decayed with an approximate half-life of 40 minutes (Fig. 2c). Following 90 min oxygen exposure, addition of 2-fold excess of prFMNH_2_ led to significant recovery of activity and demonstrates that the oxygen inactivation of VdcCD occurs at the level of the cofactor. In the case of phthaloyl co-A decarboxylase, it is postulated oxidation of the prFMN associated metal ion (Fe(II) in this case) leads to a simple loss of the cofactor^27^. However, it is unlikely oxidation of Mn^2+^ underpins oxygen inactivation of VdcCD as the addition of cofactor alone (without additional Mn^2+^) is sufficient in restoring activity.

### VdcCD crystal structure reveals a tight association between both subunits

**Fig. 3:**
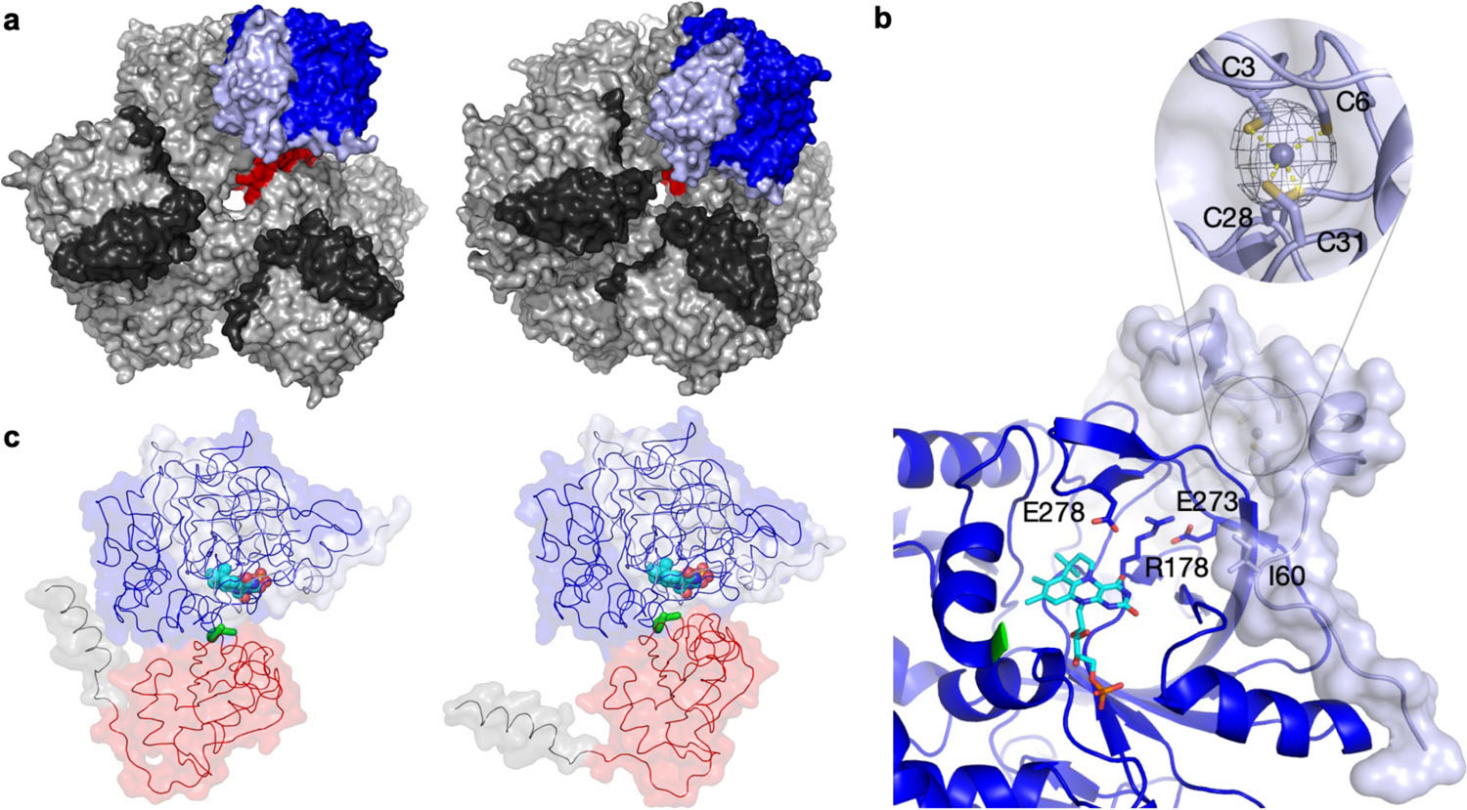
Structures of VdcCD complexes in open and closed states. **a** Left – Open hexamer of *Sh*VdcCD, Right – closed hexamer of *Sh*VdcCD. A single monomer of *Sh*VdcC is coloured by domain (red – oligomerisation domain, blue – prFMN binding domain) with *Sh*VdcD in light blue. All other *Sh*VdcC in the complex are shown in light grey, with *Sh*VdcD shown in dark grey. **b** Interaction of *Sh*VdcC and *Sh*VdcD showing the proximity of VdcD binding to the VdcC active site. VdcD interacts with loops containing the key ERE residues which are a key triad for catalysis, VdcD likely serves to maintain the positioning of these residues. prFMN (in cyan) is modelled in the active site based on a structural alignment with *holo*-Fdc1 (4ZA4). Inset shows the VdcD tetrahedral binding of Zn^2+^ by four conserved cysteine residues. Polder map corresponding to Zn^2+^ contoured at 5 sigma. **c** Dyndom analysis reveals a 32° rotation at the T325 and S326 hinge region (in green) connects the open (left) and closed states (right). Other domains coloured as in **a**, with the C-terminal helix coloured in grey.

The *apo*-*Sh*VdcCD complex was crystallised under a number of conditions, with different crystal forms yielding diffraction data at a resolution ranging from 2.2 Å to 3.4 Å. Despite multiple attempts, no crystals of individual subunits or the *holo*-*Sh*VdcCD complex could be obtained. The *Sh*VdcCD structures were solved using molecular replacement with individual domains of the *E. coli* (K12) UbiD as the search models (PDB code 5M1C). The *Sh*VdcCD crystal structures contain a *Sh*VdcC hexamer decorated with 6 small *Sh*VdcD subunits (Fig. 3a). The *Sh*VdcD was found to bind a zinc ion in a tetrahedral coordination with four cysteine residues (*Sh*VdcD numbering C3, C6, C28 and C31) (Fig. 3b). The 4 Cys residues are arranged in two CXXC knuckle motifs, an arrangement common for zinc ribbon proteins^28^. Indeed, *Sh*VdcD forms a three stranded, antiparallel β-sheet, and shares structural similarities with the zinc ribbon protein TF-IIS as identified by PDBeFold^29^ (RMSD – 1.77 Å over 37 residues - PDB accession-1TFI^30^). Both the β-sheet of *Sh*VdcD as well as the proline rich C-terminal tail region interact with the surface of *Sh*VdcC. The *Sh*VdcC:D interface was analysed by PISA^31^ and was shown to have an average area of 1661 Å^2^, involving 40 residues of *Sh*VdcD (59% of total residues), and 50 residues of *Sh*VdcC (10.5% of total residues). In addition to a number of hydrogen bonds and van der Waals interactions, intersubunit salt bridges were identified with *Sh*VdcD R12, R36 and E39 forming interactions with *Sh*VdcC D173, E31 and R133. Sequence analysis of multiple VdcD show that the two CXXC motifs are conserved, therefore Zn^2+^ binding is likely an essential structural motif to maintain the sheet formation and interaction with *Sh*VdcC. The location of the *Sh*VdcC: *Sh*VdcD interface suggests that the allosteric effect is based on maintaining the structure of the key catalytic ERE motif^32^ (Fig. 3b). Intriguingly, despite the relatively low sequence identity of 39% (48% similarity) between the VdcD subunits, limited activation of the C subunit could be achieved with the addition of a non-cognate D subunit (Fig. 2a).

### Distinct VdcCD crystal structures reveal prFMN domain motion

A comparison of the *Sh*VdcC monomers across the *Sh*VdcCD crystal structures obtained reveals that the prFMN binding domain adopts different conformations with respect to the oligomerisation domain, confirming the existence of distinct open and closed states (Supplementary movie 1 illustrates the motion between the open and closed hexamers). The distinct states occur independent of both prFMN cofactor and VdcD binding (both states occur for the *apo-*VdcCD complex), in contrast to the *apo*-open and *holo*-closed PA0254 structures where domain motion appears linked to cofactor binding^19^. *Sh*VdcCD structures thus allow for detailed inspection of the transition between the two states. An analysis of the domain motion using DynDom^33^ identifies this as a hinge motion, with the point of rotation located at T325 and S326, located in an α-helix connecting the oligomerisation and prFMN binding domains (Fig. 3c). This helix has been previously postulated as the point of flexibility in *E. coli* UbiD^20^ and AroY^18^ on the basis of the small hinge motions observed by comparing various open state monomers. In the case of PA0254, overlays of the respective *apo*- and *holo*-structures also requires a similar hinge motion^19^. In VdcCD, the hinge rotation angle is approximately 32°, with only a negligible translation of the domains (<1 Å). The number of interdomain contacts of the closed state outnumber those of the open state, with 11 more paired residue contacts, the majority occurring close to the active site (Supplementary Fig. 2).

The distance between the centre-of-mass (calculated by the CALCOM server^34^) of residues R168/173 (*Sh*VdcC/Fdc numbering) on the prFMN binding domain and L425/439 on the oligomerisation domain can be used as a simple indicator of open or closed conformation (Supplementary Fig. 3a). These residues are highly conserved in the UbiD family and have been demonstrated to be essential in catalysis by Fdc1^17,32^. In the open *Sh*VdcCD the mean distances (±SD) are 14.5 ± 0.1 Å (P1 crystal form) and 14.1 ± 1.4 Å (P2_1_2_1_2_1_ crystal form), compared to 9.3 ± 0.1 Å for the closed (F222) form. When determining the distance between the corresponding residues in other UbiD structures, only Fdc1 and *holo-*PA0254 crystal structures clearly correlate to a closed state (9.8 Å and 10.1 ± 0.1 Å respectively), while distances for AroY, UbiD, HmfF and TtnD are distributed over a wide range corresponding to the open conformation (Supplementary Fig. 3b).

### Modelling of *holo*-VdcCD complexes

**Fig. 4:**
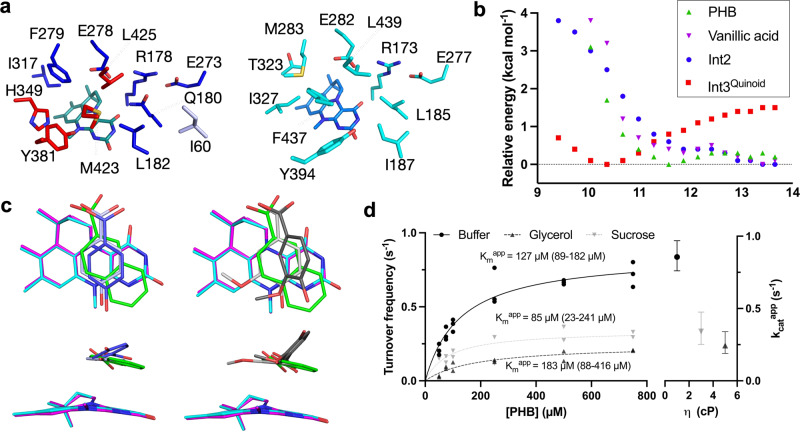
Active site architecture and the effect of domain motion. **a** Left: Active site composition of closed *Sh*VdcCD shows limited direct contribution of VdcD (I60) to the active site architecture. Residues coloured by domain as in Fig. 3. prFMN modelled in dark green from Rosetta Ligand Docking. Right: Juxtaposition of Fdc1 active site shows similar overall architecture to the active site, with residues key for Fdc1 catalysis (E282, R173, E277, L439) conserved between the enzymes. **b** Energy profile of docking models along the trajectory of domain closure. Energy minima of PHB and vanillic acid binding to VdcCD with previously docked prFMN are found when R-L distances are 11.6 and 13.4 Å respectively. **Int2** shows lowest binding energies to most open models, whereas **Int3**^**Quinoid**^ shows lowest binding energies at an R-L distance of 10 Å. **c** Modelled substrate binding modes identified by docking with AutoDock Vina. Bottom images 90° rotation about X axis. Cyan prFMN – Fdc1 (6TIB), magenta prFMN – *Sh*VdcCD Rosetta ligand docking to closed structure. Left: PHB lowest energy binding (dark blue) found when R-L distance is 11.6 Å, and binding mode (light blue) most similar to naphthoic acid (green) in Fdc1 found when R-L distance is 10.4 Å. Right: Vanillic acid lowest energy binding (dark grey) found when R-L distance is 13.4 Å, and binding mode (light grey) most similar to naphthoic acid (green) in Fdc1 found when R-L distance is 10 Å. **d** Kinetic solvent viscosity effects observed for PHB decarboxylation performed in 45% glycerol or 40% sucrose compared to buffer without viscosogen. Non-linear regression fit to Michaelis-Menten kinetics using Prism 9. K_m_^app^ does not vary significantly between samples (within 95% confidence limits). *n* = 3 (buffer) *n* = 2 (glycerol and sucrose) technical replicates, all replicates shown. Similar trends were seen in two independent replicates. The inset shows calculated k_cat_^app^ plotted against relative viscosity (η) revealing a significant decrease in catalytic rate constant with increasing viscosity. Values of η estimated from literature^51^. Data shown as mean, with error bars showing 95% confidence limits.

An overlay of the structure of a closed *Sh*VdcCD structure with the model UbiD enzyme *Aspergillus niger* Fdc1 (PDB 4ZA4), allows for the *Sh*VdcCD prFMN binding site to be easily located and active site residues identified. Docking prFMN to the closed *Sh*VdcCD structure using Molsoft ICM Pro (version 3.9-2b) and Rosetta Ligand Docking^35,36^ resulted in a similar binding conformation (Fig. 4a). All residues implicated in catalysis, including the UbiD-conserved E(D)RE motif integral for activity, are present in *Sh*VdcCD.

We were able to assess binding of vanillic acid in *Sh*VdcCD by superimposition with the naphthoic acid (6TIB) *An*Fdc complex^5^ (Supplementary Fig. 4). The proposed substrate binding mode suggests H349 and Y381 position the substrate of the Cα-Cβ bond in ideal geometry in relation to C1’ and C4a and could support substrate phenolate formation during catalysis. To assess the impact of domain motion on catalysis, we assessed the binding of substrate and prFMN adducts to intermediate structures along the trajectory of closure (generated using Chimera Morph between the open and closed states^37^) by docking using AutoDock Vina^38^. The morph model used for docking was validated by assessing the domain motion using low-frequency normal mode analysis performed with elNémo^39^ (Supplementary movie 2), which shows similar domain motions. The catalytic glutamate residue (E278) adopts a range of conformations in other UbiD enzymes, adapting to the presence and nature of ligands bound in the active site. For this reason, the E278 rotamer was altered to avoid clashes when docking carboxylated compounds. Docking PHB to the *holo-*VdcCD reveals intermediate states (i.e. R-L distance = 11.6 Å) result in lowest energy, whereas docking vanillic acid reveal that R-L distances of 13.4 Å show the lowest energy (Fig. 4b). However, the relative position of the substrate with respect to prFMN is subtly altered by the domain closure, and resembles previously determined Fdc1 ligand complexes most closely at R-L distances of 10.4 Å and 10.0 Å for PHB and vanillic acid respectively (Fig. 4c). Hence, domain closure likely minimizes the substrate Cα/prFMN C1’ distance prior to **Int1** formation.

**Fig. 5:**
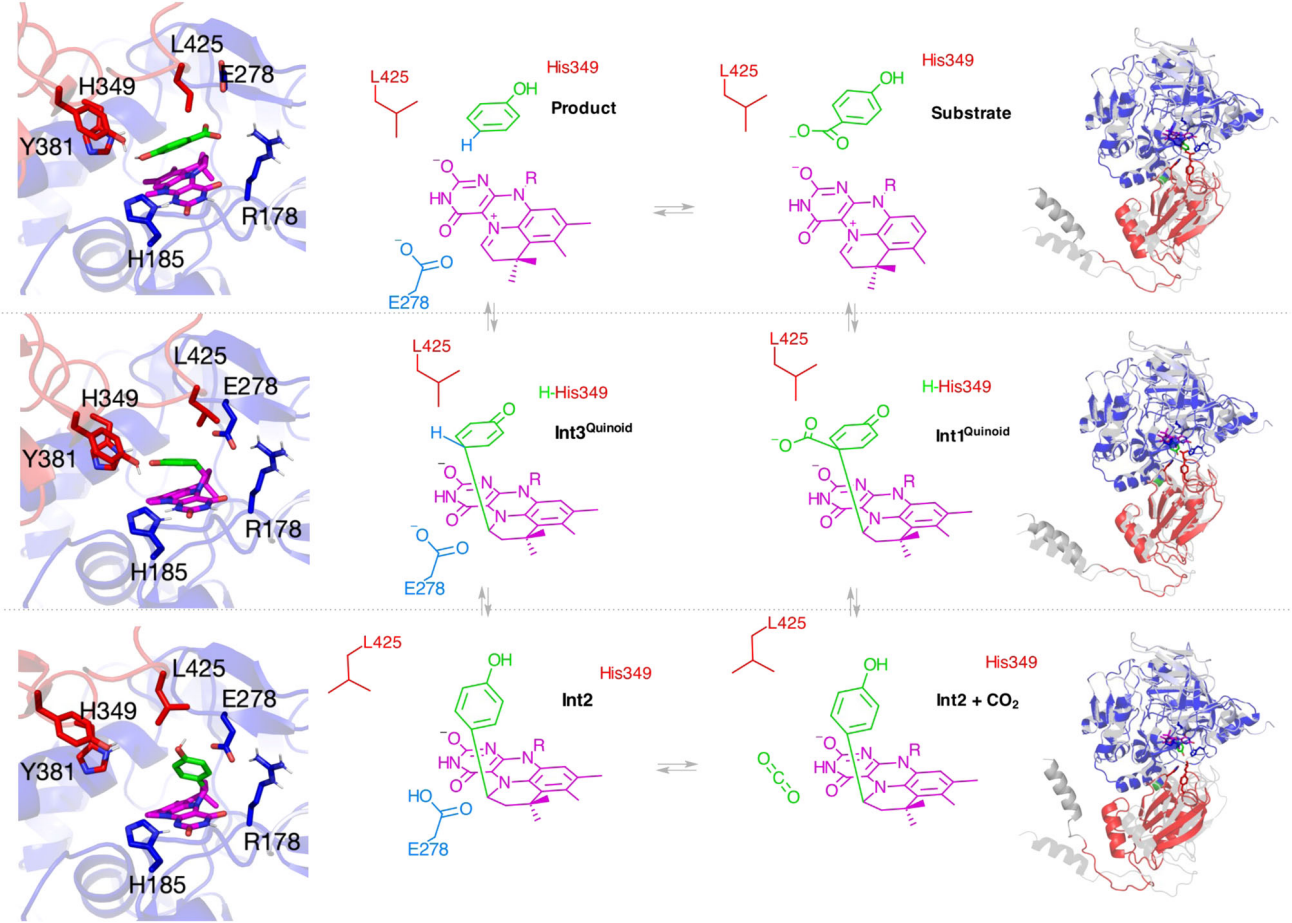
VdcCD catalysis requires large scale domain motion. Proposed VdcCD mechanism shown with structures to the left showing the lowest energy configuration of substrate and intermediates bound in *Sh*VdcCD active site (prFMN depicted in magenta, and substrate/adducts depicted in green). The structures to the right show the corresponding monomer conformation, overlayed on the closed structure (in translucent grey). Supplementary movie 3 illustrates the domain motions seen between intermediates.

DFT generated models of the PHB adducts **Int2** and **Int3**^**Quinoid**^ (**Int1**^**Quinoid**^ spontaneously decarboxylates) were docked to *apo*-VdcCD structures along the domain closure trajectory. The **Int2** complex has the lowest energy for the longer R-L distances, while **Int3**^**Quinoid**^ complex displays an energy minimum at R-L distance ~10 Å (Figs. 4b and 5). Hence, docking analysis suggests domain motion accompanies the **Int 1/3**^**Quinoid**^ transition to **Int2**. Furthermore, it is possible domain closure leads to a strained conformation of **Int2**, thus assisting conversion to **Int1/3**^**Quinoid**^ and ultimately product formation.

### Kinetic solvent viscosity effects support a VdcCD dynamic model

To determine whether catalysis is coupled to proposed domain motions, we determined the kinetic parameters of PHB decarboxylation by *Sh*VdcCD in the presence of viscosogens. It was found that k_cat_^app^ was drastically reduced in 45% (v/v) glycerol or 40% (w/v) sucrose conditions (Fig. 4d). In contrast, the K_m_^app^ for PHB is not significantly impacted within the error of the data, suggesting neither microviscosogen acts as competitive inhibitor. The decrease of k_cat_^app^ with relative viscosity (η) supports the proposed VdcCD dynamic model for catalysis with increased viscosity affecting domain motion^40^.

### The VdcD unit does not directly contribute to catalysis

Only a single *Sh*VdcD residue (I60) can be considered to contribute to the active site architecture (Figs. 3b and 4a). This residue is located on a conserved proline rich tail located at the *Sh*VdcCD interface. We sought to investigate the role of this C-terminal tail by deleting *Sh*VdcD residues 59–68 (VIPPIPPLKK). The resulting *Sh*VdcCD^V59X^ complex was determined to be active, with decarboxylation yields only slightly diminished (i.e. <10% reduction in yield) compared to those of the wild type (Fig. 2a). *Sh*VdcCD^V59X^ crystallised in a different space group (P2_1_2_1_2_1_) from the WT open (P1) and closed (F222) forms. The *Sh*VdcCD^V59X^ P2_1_2_1_2_1_ structure contains a single hetero-dodecamer in the asymmetric unit, with the majority of individual *Sh*VdcC monomers adopting an open form. A comparison to the wild type structure shows little difference in the relative positions of the active site residues, with the exception of a minor shift in residues directly adjacent to the deleted tail region. The observation that *Sh*VdcD does not appear to directly contribute to the active site architecture supports the notion that its role is mainly in the stabilisation of the catalytic ERE containing loop regions at the *Sh*VdcCD interface.

### VdcCD substrate specificity

The substrate scope of both *Bs*VdcCD and *Sh*VdcCD were determined by endpoint assays, incubating the enzymes overnight with 5 mM substrate prior to quenching the reaction. The substrate scope of these enzymes is shown to be similar, as expected given the 58% identity between *Sh*VdcCD and *Bs*VdcCD (Fig. 2a). Notable exceptions are syringic acid, for which product formation could only be detected in the presence of *Bs*VdcCD, and gallic acid which only supported minor activity with *Sh*VdcCD. This can be explained by consideration of the respective active site structures. The *Bs*VdcCD active site volume is larger due to the substitution of Met423 for Leu419 and Leu182 for Val179 on the C subunit, as well as the D subunit Ile60 replaced by Val67 (Supplementary Fig. 5). This presumably allows for correct positioning of the second methoxy group of syringic acid. An L182V mutant of *Sh*VdcCD was found to be capable of syringic acid decarboxylation with 6.5 ± 0.6% turnover compared to no turnover seen in the wild type protein, thus further validating the proposed substrate binding model.

## Discussion

The covalent catalysis used by the prFMN-dependent enzymes presents a range of challenges to ensure rapid turnover. In the case of the fungal Fdc1 enzyme, considerable strain imposed by the rigid active site for various intermediates along the pathway has been observed^17^. This is proposed to support the reversible nature of the 1,3-dipolar cycloaddition process and avoid the formation of dead-end adduct species. While these are avoided for the relatively large substrates, dead-end adducts are formed with the considerably smaller crotonic acid, presumably because the enzyme is unable to exert strain in this case. The aromatic acid UbiD decarboxylases are faced with a range of challenges. These including the need to surmount the inherent aromatic stability of the substrate, the requirement to accommodate the relatively large motion of the substrate aromatic moiety upon progression through the various prFMN-covalent adducts, as well as the ability to manipulate the internal thermodynamics of the reaction to avoid highly stable aromatic **Int2** species following decarboxylation/deprotonation.

We demonstrate that UbiD-enzymes have the scope to adapt the shape of the active site via domain motion by determining structures of open and closed states for the vanillic acid decarboxylase VdcCD (Fig. 5). While the VdcCD closed state resembles the conformation observed for fungal Fdc1, the open state resembles the majority of UbiD structures hitherto determined. Modelling of the VdcCD domain closure trajectory and assessing the binding of substrate and intermediates reveals an intermediate conformation binds substrate most favourably. Further domain closure effectively brings prFMN and substrate together and favours binding of the **Int1/Int3**^**Quinoid**^ states. The *sp*^3^ to *sp*^2^ transition of the substrate Cα concomitant with decarboxylation can be accommodated by opening of the active site. Re-aromatisation thus drives formation of the highly stable **Int2** species and domain opening. If the energetics of domain closure are such that the open-closed equilibrium is poised towards the closed state, this would provide a means to exert strain on **Int2** by domain closure and avoid formation of dead-end intermediates. The kinetic solvent viscosity effects observed for PHB decarboxylation align with the proposed dynamic model for VdcCD catalysis. It is likely that (de)carboxylation of other aromatic acids, although not necessarily progressing through a quinoid intermediate, requires similar active site mobility to accompany formation of and interconversion between covalent prFMN adducts.

UbiD enzyme activity is often found to be oxygen sensitive, with the exception of family members acting upon acrylic (Fdc1) or heteroaromatic substrates (PA0254). Both Fdc1 and PA0254 form dimers^16,41^, in contrast to the more frequently observed hexameric nature of the aromatic acid decarboxylases^18,20^. Furthermore, there is little evidence that large scale domain motion occurs for the dimeric *holo*-enzymes or is involved in catalysis. In contrast, based on our work with VdcCD, it appears domain motion is integral to enzyme turnover for aromatic acid (de)carboxylation. It is possible the open state preferentially populated by the hexameric *holo*-enzymes is markedly different in oxygen sensitivity compared to the closed state readily observed for the dimeric UbiD enzymes. Furthermore, the prFMN reactivity is likely to be modulated by the protein environment, in particular residues that interact with the O1 position. In the case of the oxygen sensitive UbiD enzymes, these include positively charged residues (i.e. Arg/Lys/His), while for those apparently unaffected by oxygen, hydrogen bonding partners are Asn or Gln residues. This difference in active site composition may be responsible for the ability to decarboxylate acids fused directly to the aromatic ring, at the expense of stability in aerobic conditions. Indeed, evolved variants of the oxygen tolerant Fdc1 have been reported to undergo oxygen mediated inactivation^10^, suggesting active site properties influence oxygen sensitivity.

The rational engineering of *Sh*VdcCD to accept syringic acid using L182V provides further evidence that UbiD enzymes can be evolved, but also suggests further studies will need to take into account the effect mutations might have on the protein dynamical behaviour. In conclusion, domain dynamics coupled to catalysis provides a solution to some of the challenges faced by aromatic acid UbiD decarboxylases. Appropriate choreography between domain dynamics and catalysis is likely essential to achieve rapid turnover, by accommodating large scale motion of the substrate aromatic moiety and manipulating the internal thermodynamics to avoid the pitfall of an excessively stable **Int2** species. This certainly would be the case of very challenging substrates such as benzene or naphthalene^14,15^. Indeed, while rational engineering of the dimeric Fdc1 has led to some success in expanding the substrate scope, Fdc1 mediated naphthoic acid decarboxylation remains very slow^5^. It seems likely the lack of significant domain motion in Fdc1 will need to be addressed in order to achieve improved catalytic rates.

## Methods

### Cloning, mutagenesis, and cell growth

*Sedimentibacter hydroxybenzoicus vdcC* and *vdcD* genes were codon optimized for *E. coli* expression and synthesised by GeneArt (ThermoFisher), *Bacillus subtilis vdcC* and *vdcD* were codon optimized for *E. coli* expression and synthesised by GenScript. C-terminal His tag constructs of the C subunits were made in pET30a between *NdeI* and *XhoI* restriction sites. N-terminal His tag constructs of D subunits were made in pET28a between *NdeI* and *XhoI* restriction sites. Untagged constructs of both C and D subunits were made in pET21a between *NdeI* and *XhoI*. A construct of untagged *Pa*UbiX in pCDF-Duet was used^6^. These constructs were made to allow different combinations of vectors to be used with different antibiotic resistances to maintain selection pressures.

The genes were amplified using the PCR primers detailed in Supplementary Table 2.

The empty vectors were cut using restriction enzymes purchased from NEB. In-Fusion (Takara) ligation independent cloning was used to insert the fragments into cut vectors. Stellar cells (Takara) were transformed with the product, and insertion was confirmed by sequencing (Eurofins) following MiniPrep (Qiagen) of overnight cultures of single colonies.

Mutants were produced by using Q5 mutagenesis (NEB) as per the manufacturers protocols, using the primers in Supplementary Table 2. Mutation was confirmed by sequencing.

BL21(DE3) cells (NEB) were transformed with plasmid in the combinations denoted in Supplementary Table 3.

BL21(DE3) strains were grown at 37 °C, 180 rpm shaking in 1 L conical flasks of Terrific Broth (Formedium) with 0.4% (v/v) glycerol added, along with 50 mg L^−1^ of the appropriate antibiotic(s). Cells were induced with 0.25 mM IPTG when OD_600nm_ reached 0.8, at which point the temperature was reduced to 18 °C overnight. Cells were harvested after typically 18 h of induction by centrifugation at 7000 × *g* for 10 min in a Beckman JLA8.1000 rotor.

### Purification

Cells were resuspended in buffer A (50 mM Hepes, 300 mM NaCl, pH 7.5) with SigmaFAST EDTA free protease inhibitor cocktail tablet added, 0.1 mg mL^−1^ DNase and RNase were both added to the cell suspension (all Sigma). Cells were lysed using a Constant Systems cell disruptor at 20 kPsi. Lysate was clarified by centrifugation at an average 184,000 × *g* for 1 h in a Beckman Type 50.2Ti rotor. Using an AKTA Pure (GE Healthcare), the lysate was applied to a pre-equilibrated 5 mL HisTrap-HP (GE Healthcare) column and was washed with 5 CV buffer A. A gradient of buffer A to buffer A containing 250 mM imidazole was run over 30 CV and collected in 1 mL fractions. Proteins eluted in a single peak and were run on an SDS PAGE gel for confirmation of purity. Fractions containing the desired protein were concentrated using Sartorius Vivaspin concentrators, and subjected to gel filtration using a Superdex 200 10/300 GL column (GE Healthcare), in 20 mM Hepes, 150 mM NaCl, pH 7.5 to remove any excess VdcD and to ensure 1:1 stoichiometry.

For protein prepared for crystallisation, the *Sh*VdcCD^N^/*Sh*VdcCD^V59X^ complexes were further purified after IMAC. The fractions from Ni-IMAC were pooled and concentrated, prior to a 6-fold dilution using 50 mM Hepes, pH 7.5 to dilute NaCl to 50 mM. Using an AKTA Pure (GE Healthcare), the complex was applied to a 5 mL HiTrapQ-HP (GE Healthcare) column and was washed with 5 CV 50 mM Hepes, 50 mM NaCl, pH 7.5. A gradient of 50 mM − 1 M NaCl was run over 30 CV, in a constant buffer of 50 mM Hepes pH 7.5. The peak containing the complex was pooled, concentrated, and subjected to gel filtration using a Superdex 200 10/300 GL column (GE Healthcare), in 20 mM Hepes, 150 mM NaCl, pH 7.5. The protein was concentrated in Vivaspin concentrators (Sartorius) and crystal trials were immediately performed.

Protein concentrations were estimated using A_280nm_ extinction coefficients calculated by the ExPASy ProtParam tool: *Sh*VdcC ε = 57300 M^−1 ^cm^−1^, *Bs*VdcC ε = 58790 M^−1 ^cm^−1^, *Sh*VdcD, *Sh*VdcD^V59X^, and *Bs*VdcD ε = 13980 M^−1 ^cm^−1^, *Sh*VdcCD ε = 71280 M^−1 ^cm^−1^, *Bs*VdcCD ε = 72770 M^−1 ^cm^−1^.

### HPLC activity assays

All HPLC assays were prepared in an anoxic chamber (Belle Technologies) in a nitrogen atmosphere. prFMN was made as per previous protocols^42^; 50 µM UbiX^11^, 5 µM Fre reductase^43^, 1 mM FMN, 5 mM DMAP, and 5 mM NADH were combined in an anaerobic chamber and left for 2 h to allow prFMN production before filtration through a 10 kDa MWCO centrifugal concentrator (Sartorius) to remove UbiX and Fre proteins prior to addition to Vdc assays.

Initial assays were performed with substrates (4-hydroxybenzoic acid (PHB) and vanillic acid) dissolved to 10 mM in 100 mM KP*i*, pH 6; substrates were not purged of oxygen before introduction to the anaerobic chamber. VdcC, VdcD or both were mixed with prFMN and MnCl_2_ in a 1:2:2 (protein: prFMN: MnCl_2_) in an anaerobic chamber and were added to substrate to a final concentration of 10 µM protein (both VdcC and VdcD 10 µM when added in combination), and diluted to a final substrate concentration of 5 mM. The reaction was allowed to proceed overnight (18 h) in the anaerobic chamber before being removed to aerobic conditions and quenched by the addition of 1 volume equivalent of acetonitrile + 0.2% trifluoroacetic acid (TFA). The samples were centrifuged at 14,000 × *g* for 10 min to removed precipitated protein. The resulting supernatant was diluted 2.5 fold before being subjected to HPLC in isocratic 50% acetonitrile, 50% water, 0.1% TFA on a Phenomenex Kinetex 5 µm C18 100 Å 250 × 4.6 mm LC Column. Peaks were identified by running standards of substrate and expected product and quantified by running a standard concentration curve of these compounds. A control of substrate with 20 µM prFMN was made to ensure there were no side reactions arising from the prFMN reconstitution mix. Samples were run in duplicate.

Assays for the substrate scope experiments were set up in an identical manner, with the exception of adding a 2-fold excess of VdcD to VdcC in cross-activation studies. In all assays the concentration of the C subunit was maintained at 10 µM.

Data were analysed and peaks integrated using Agilent Chemstation (Version (B.04.03).

### VdcC:VdcD Stoichiometry effect assays

The effect of varied stoichiometry was investigated by the anaerobic reconstitution of *Sh*VdcC in the presence of 0.2 and 4 molar equivalents of *Sh*VdcD. *Sh*VdcC and *Sh*VdcD were mixed with prFMN/MnCl_2_, giving constant concentrations of 50 µM and 200 µM for *Sh*VdcC and prFMN/MnCl_2_ respectively, and a concentration of either 10 µM or 200 µM for *Sh*VdcD. The activity was assayed in anoxic conditions on a Cary 60 UV–vis Spectrophotometer (Agilent) using the Kinetics software (Version 5.0.0.999) measuring the change in absorbance at 247 nm using 100 µM PHB, and adding VdcC to a concentration of 0.25 µM. Substrate was not purged of oxygen prior to activity assays. The measurements were taken over a period of 3 h in triplicate.

### Aerobic decay

To measure the aerobic activity decay, *Sh*VdcCD was first rendered anaerobic by desalting into 20 mM Hepes, 150 mM KCl, pH 7.5 in an anaerobic chamber using a GE Healthcare PD-10 Mini desalting column. prFMN (produced as described above) and MnCl_2_ were added in 2 fold excess. The mixture was left for 10 min prior to desalting into the same buffer. A total of 100 µL of active protein was removed from the anaerobic environment in an opaque tube (to ensure no light mediated effects) and the activity was measured every 15 min over a 90 min period using 100 µM PHB in 50 mM NaPi, 50 mM KCl, pH 6, measuring the decrease in absorbance at 247 nm in a Agilent Cary 60 Spectrophotometer using the Kinetics software (Version 5.0.0.999). The protein was constantly exposed to oxygen by leaving the tube open throughout the time course. After 90 minutes the remaining protein was reintroduced to the anaerobic chamber, and a 2-fold excess of prFMN was added. The protein was removed from the chamber, and immediately assayed for activity. The process was performed in duplicate.

### Kinetic solvent viscosity effects (KSVEs)

To measure KSVEs, PHB was dissolved in 50 mM NaP*i*, 50 mM KCl, pH 6, and added to stock solutions of buffered viscosogen (50 mM NaPi, 50 mM KCl, pH 6) to a final PHB concentration of 10 mM, and viscosogen concentrations of 45% glycerol and 40% sucrose. The pH of PHB/viscosogen stock solutions were measured to ensure no variation from pH 6. The PHB stocks were mixed with buffered viscosogen to generate the dilution series required for obtaining kinetic parameters.

prFMN was produced in vitro as described above, and was added to *Sh*VdcCD in a substoichiometric ratio with excess MnCl_2_ (0.5 prFMN: 1 *Sh*VdcCD: 2 MnCl_2_). This was performed so the final concentration of prFMN (assuming complete turnover in the UbiX reaction) can be considered E_t_ for calculations (i.e. [active protein] = [prFMN]). The mixture was diluted to E_t_^app^ = 30 µM with a mixture of aerobic and anaerobic buffers (1:3 ratio) and left overnight in the anaerobic chamber to allow full oxidative maturation in trace oxygen.

Rates were measured using the decrease in absorbance at 247 nm in an Agilent Cary 60 Spectrophotometer in the Kinetics software (Version 5.0.0.999), using Eppendorf UVette^®^ cuvettes. While measuring rates at [PHB] ≤100 µM, a 1 cm pathlength was used; at ≥250 µM a 0.2 cm pathlength was used to allow absorbance in the linear range. *Sh*VdcCD^prFMN^ was added to 100 µL PHB and agitated vigorously.

### Crystallography

Crystal trials were attempted with both *Bs*VdcCD and *Sh*VdcCD following purification. Trials were produced in Molecular Dimensions’ PACT-Premier, JCSG-Plus, Morpheus, SG1, LMB, and BCS screens. Using a TTP Labtech Mosquito, protein was mixed in a 1:1 ratio with reagent, in 600 nL sitting drops with 30 µL reservoir solution. Three concentrations of protein were screened in SWISSCI 3 Lens plates, at 20, 10, and 5 mg mL^−1^. *Bs*VdcCD was found to crystallise in a number of conditions, however, diffraction was insufficient. *Sh*VdcCD was found to crystallise in multiple conditions, with the best diffracting crystals found in JCSG-Plus D3 (0.2 M Sodium chloride, 0.1 M sodium/potassium phosphate, pH 6.2, 50% v/v PEG200) (F222-Closed), BCS H2 (0.1 M Hepes, pH 7.5, 30% v/v PEG Smear low) (P1-Open), and JCSG-Plus B8 (0.2 M Magnesium chloride hexahydrate, 0.1 M Tris, pH 7, 10% PEG 8000) (P2_1_2_1_2_1_-*Sh*VdcCD^V59X^). The *Sh*VdcCD^V59X^ crystal was cryoprotected with 10% PEG 400 prior to flash cooling in liquid N_2_. F222 and P1 wild type *Sh*VdcCD crystals were flash cooled and required no further cryoprotecting due to components in the crystallisation conditions. Data were collected on Diamond Light source beamlines i03 (F222), i04-1 (P1), and i24 (V59X) and processed with xia2^44^ (P1 and F222) or xia2 dials^45^(P2_1_2_1_2_1_) using beamline automated data processing. Structures were solved using molecular replacement, using Phaser MR^46^, with UbiD as a search model. Two ensembles were used to solve the structure, firstly the hexameric core of the oligomerisation domains was used as a single search model (UbiD residues 334–465 arranged in the hexamer), followed by the search for six prFMN binding domains (UbiD residues 1–333). The F222 model was initially built using phenix.autobuild^47^, prior to manual model building in Coot^48^. The model was refined using phenix.refine^49^, interspersed with manual model building. The closed model was then used in molecular replacement of the open structures (P1, P2_1_2_1_2_1_), using the same strategy as was used for solving the closed structure. The models were then refined and built using phenix.refine and coot. The models and data were deposited in the PDB under the accession codes (Closed–7AE4, Open–7AE5, *Sh*VdcCD^V59X^–7AE7). Data processing statistics, along with refinement statistics are in Table 1.

**Table 1:** X-ray crystallography data collection and refinement statistics.

	7AE4 (Closed)	7AE5 (Open)	7AE7 (V59X)
Wavelength (Å)	1.284	0.9159	0.9686
Resolution range (Å)	64.05−3.31 (3.428−3.31)	63.87−2.19 (2.268−2.19)	71.23−2.66 (2.755−2.66)
Space group	F 2 2 2	P 1	P 21 21 21
Unit cell Å °	270.92 320.55 326.23 90 90 90	98.23 108.3 111.42 117.72 101.32 91.8	101.553 200.945 201.991 90 90 90
Total reflections	783105 (80789)	350629 (34881)	2553323 (157443)
Unique reflections	104765 (10415)	197121 (19440)	119016 (11673)
Multiplicity	7.5 (7.8)	1.8 (1.8)	21.5 (13.5)
Completeness (%)	99.94 (99.98)	97.03 (95.74)	99.85 (98.96)
Mean I/sigma(I)	13.95 (1.52)	6.59 (1.42)	8.25 (2.79)
Wilson B-factor Å^2^	126.16	43.17	37.25
R-merge	0.1062 (1.554)	0.05937 (0.563)	0.3177 (0.845)
R-meas	0.1141 (1.665)	0.08396 (0.7962)	0.3253 (0.8782)
R-pim	0.04155 (0.5936)	0.05937 (0.563)	0.06934 (0.2302)
CC1/2	0.998 (0.511)	0.984 (0.445)	0.988 (0.618)
CC*	1 (0.822)	0.996 (0.785)	0.997 (0.874)
Reflections used in refinement	104747 (10413)	197043 (19412)	118974 (11673)
Reflections used for R-free	5121 (479)	9805 (1007)	5811 (531)
R-work	0.1625 (0.2993)	0.2233 (0.3399)	0.1799 (0.2609)
R-free	0.1974 (0.3295)	0.2625 (0.3651)	0.2268 (0.3235)
CC (work)	0.942 (0.744)	0.938 (0.626)	0.926 (0.811)
CC (free)	0.932 (0.698)	0.911 (0.552)	0.929 (0.685)
Number of non-hydrogen atoms	25697	25337	24349
macromolecules	25588	24515	23802
ligands	175	18	11
solvent	None	804	536
Protein residues	3257	3192	3146
RMS (bonds) (Å)	0.004	0.003	0.03
RMS (angles) (°)	0.64	0.62	0.66
Ramachandran favored (%)	95.48	96.7	97.45
Ramachandran allowed (%)	4.52	3.27	2.51
Ramachandran outliers (%)	0	0.03	0.03
Rotamer outliers (%)	0	1.04	1.34
Clashscore	12.04	5.81	5.72
Average B-factor (Å^2^)	130.56	56.9	45.55
macromolecules	130.53	57.18	45.7
ligands	138.43	75.04	53.65
solvent	None	48.18	38.76
Number of TLS groups	1	82	1

### Modelling and docking

The prFMN binding domains of the two states were aligned on secondary structure, prior to a Morph being calculated using UCSF Chimera 1.13.1^37^. Analysis showed that the largest motions were from the closure of the domains, however, loop motions around the prFMN binding pocket were observed, with the closed state closely resembling crystal structures in which prFMN can be seen binding. Therefore, a hybrid model was constructed, with the prFMN binding domain (residues 1–323) of the closed structure being used in conjunction with the oligomerisation domain of the open structure to create an open model with the prFMN binding pocket in an appropriate conformation. A new morph was performed, with the trajectory of the oligomerisation domain being plotted in 16 models, allowing an approximate 2° iterations of the motion. Cartesian coordinates for the **Int2** and **Int3**^**Quinoid**^ intermediates with the simplest substrate (PHB) were built from previous DFT models of prFMN-substrate adducts^17^ and were optimised using Gaussian 09 revision D.01 at the B3LYP/6-311 + +G(d,p) level of theory with the D3 version of Grimme’s dispersion with Becke–Johnson damping^50^ and a generic polarizable continuum model with a dielectric of 5.7. These intermediates were docked into the 16 morph models using AutoDock Vina^38^. To ensure only the effect of domain closure was being observed, all residues were restricted to a single conformer.

Substrate and product docking was performed using the coordinates described above, with prFMN present in the position as determined by Rosetta Ligand Docking^35^. For PHB docking E278 was allocated an allowed rotamer to prevent occlusion of the substrate carboxylate binding pocket.

Docking of **Int2** and **Int3**^**Quinoid**^ resulted in single binding poses in each of the sixteen models. Where substrates and product were docked, multiple poses were observed. In these instances, the pose with the lowest energy for the correct orientation (of substrate/product carboxy and hydroxy groups based on prior knowledge) was selected to resemble a likely catalytically relevant species.

Models and ligand co-ordinates used for docking are available at 10.5281/zenodo.5101186, along with binding energies calculated by Autodock Vina.

ElNémo was used to assess the low frequency normal mode analysis of domain motion from crystal structures. Both open and closed structures were analysed. Parameters were set as default, with the exception of NRBL = 5, and DQMIN = −200, DQMAX = 200.

### Reporting summary

Further information on research design is available in the Nature Research Reporting Summary linked to this article.

## Supplementary information

Supplementary information

Description of Additional Supplementary Files

Supplementary Movie 1

Supplementary Movie 2

Supplementary Movie 3

Reporting Summary

## Data Availability

The atomic coordinates and structures factors (codes 7AE4, 7AE5, and 7AE7) have been deposited to the Protein Data Bank (http://www.pdb.org). Graphical data presented here is available in the source data file. Modelling data is available at 10.5281/zenodo.5101186. Publicly available data used in this study is available from the Protein Data Bank under the accession codes: 4ZA4, 5O3M, 5O3N, 5NY5, 5M1C, 5M1D, 6H6V, 6DA6, 6DA7, 6DA9, 7ABN Source data are provided with this paper.

## Source data

Source Data
