# Supplementary material for: UbiD domain dynamics underpins aromatic decarboxylation
Source: Nat Commun. 2021 Aug 20;12:5065. doi: 10.1038/s41467-021-25278-z (PMC8379154; doi:10.1038/s41467-021-25278-z)
Supplement: Supplementary file 1 — Supplementary information [file 41467_2021_25278_MOESM1_ESM.docx]

**UbiD domain dynamics underpins aromatic decarboxylation**

Stephen A. Marshall^1a^, Karl A. P. Payne^1^, Karl Fisher^1^, Gabriel R. Titchiner^1^, Colin Levy^1^, Sam Hay^1^, David Leys^1b^

^1^Manchester Institute of Biotechnology, University of Manchester, Manchester M1 7DN, UK

^a^ Stephen.marshall@manchester.ac.uk, ^b^ David.leys@manchester.ac.uk

**Supplementary information**

| **Enzyme (PDB ID)** | **References** | **Substrate** |
| --- | --- | --- |
| UbiD  (5M1D) | ^1-3^ |   n = species variable |
| Fdc1  (6EV4) | ^4-6^ |  |
| AroY  (5O3N) | ^7,8^ |  |
| HmfF  (6H6V) | ^9^ |  |
| PA0254  (7ABN) | ^10^ |  |
| TtnD  (6DA9) | ^11^ |  |

**Supplementary table 1: Substrates of UbiD homologues with published structures.** Carboxylate group removed during catalysis shown in red.

**Supplementary Figure 1: prFMN production and oxidation.** prFMN is produced by UbiX utilising reduced flavin mononucleotide (FMNH_2_) and dimethyl allyl mono/pyrophosphate (DMA(P)P). Reduced prFMN (prFMNH_2_) undergoes a two electron oxidation with a UbiD active site to form the active prFMN^iminium^ cofactor.


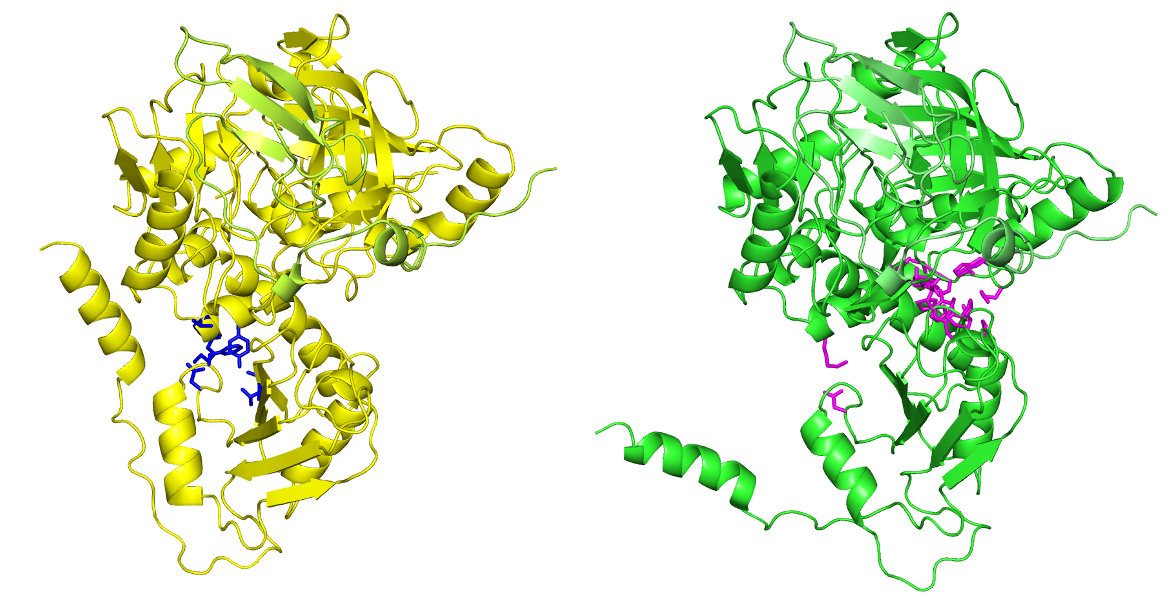


**Supplementary Figure 2: Inter-domain contacts made in open and closed states.** Left; Open state, interdomain contacts shown in blue. Right; closed state, interdomain contacts shown in magenta, located predominantly in active site cleft.


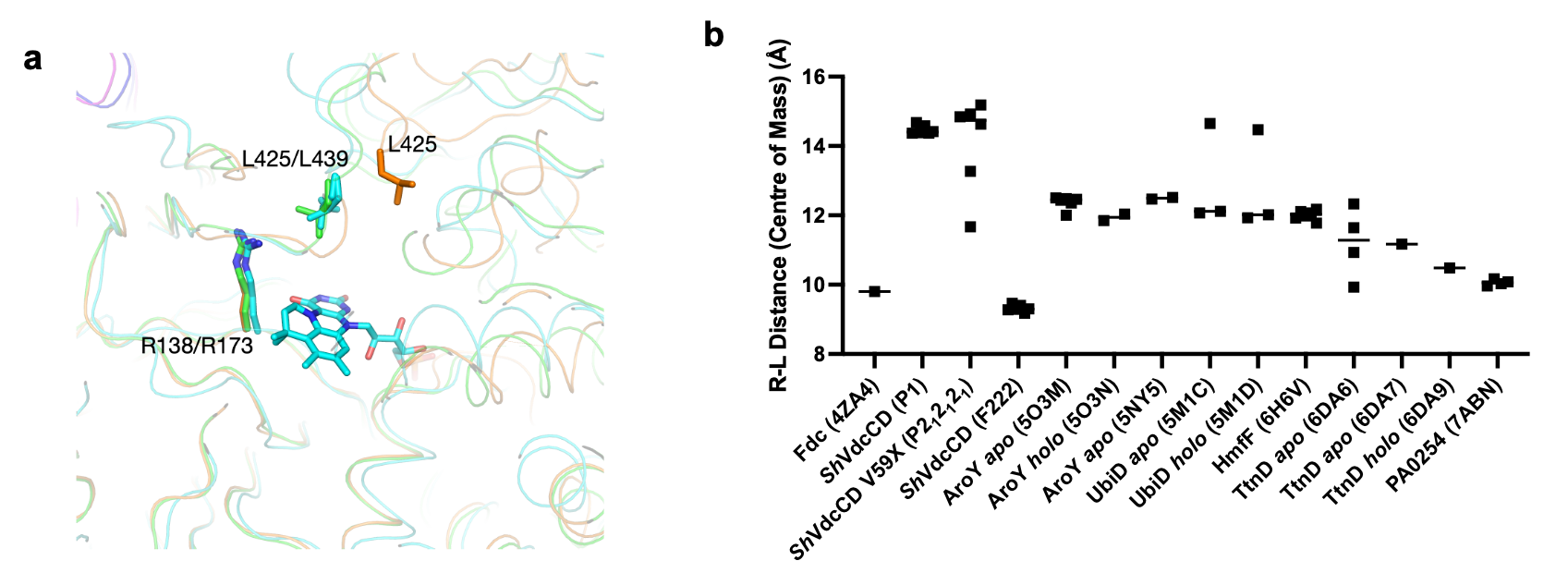


**Supplementary Figure 3: R-L Distances as an indicator of domain closure.** **a)** Overlay of Fdc1 (cyan), *Sh*VdcCD closed (green) and *Sh*VdcCD open (orange) demonstrating the distance variation between R168/173 and L425/439 (Vdc/Fdc numbering). **b)** R-L distances of solved crystal structures using the centre of mass (calculated by <http://bioinformatica.isa.cnr.it/CALCOM/introduction.html> server). Line in box is median distance, all distances from monomers in structures shown. *PA0254 possesses methionine in place of leucine. The use of centre of mass rather than Cα reduces variation due to substitutions.


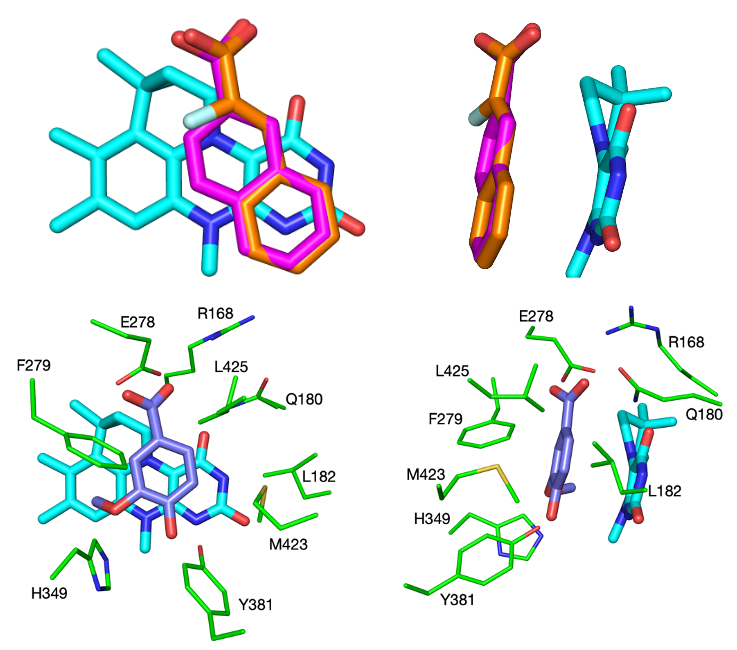


**Supplementary Figure 4: Modelling vanillic acid binding VdcCD based on Fdc substrate binding.** Top: binding of α-fluorocinnamic acid (orange) and naphthoic acid (magenta) in Fdc1 crystal structures (6R2R and 6TIB respectively). Bottom: Vanillic acid binding modelled in *Sh*VdcCD active site. Vanillic acid was modelled in VdcCD in similar binding mode with respect to prFMN as 6R2R/6TIB structures. L182 can be seen to occlude binding of substitutions at the 5 position. Right images are 90 ° rotation about Y axis.


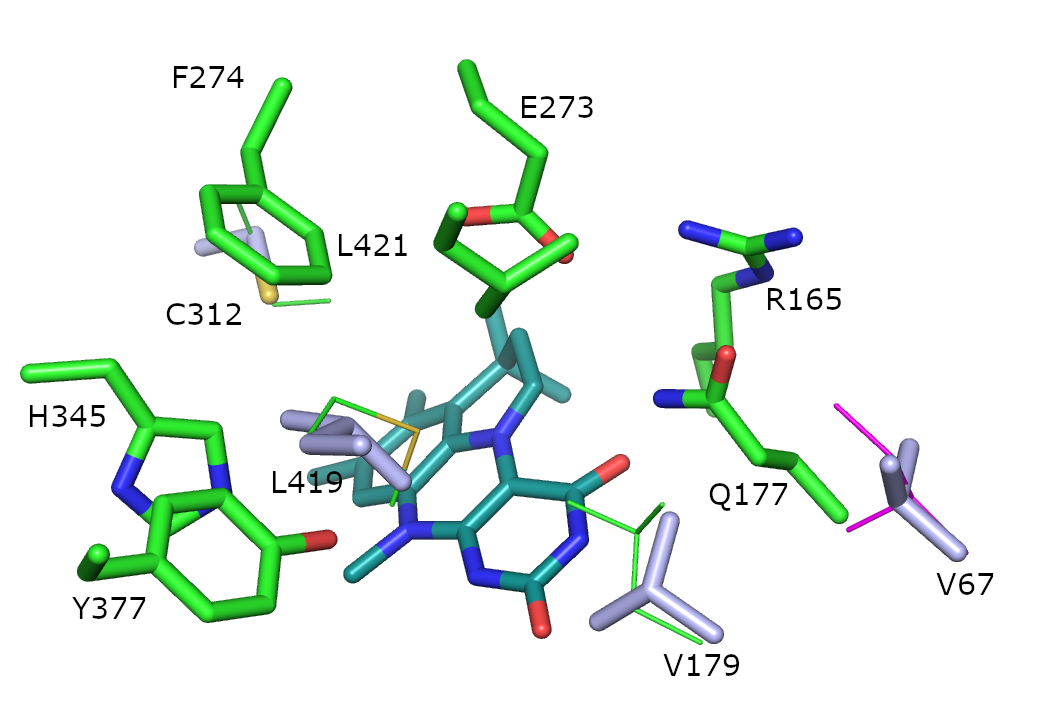


**Supplementary Figure 5: Model of closed *Bs*VdcCD determined through sequence alignment with *Sh*VdcCD**. Residues which vary from *Sh*VdcCD are shown in light blue sticks, with *Sh*VdcCD counterparts in lines. Green sticks represent unchanged residues. Numbering according to *Bs*VdcCD active site.

| Primer | Sequence (5’ – 3’) |
| --- | --- |
| *Sh*VdcC_21/30F | AAGGAGATATACATATGGCCAAAGTGTATAAAGATC |
| *Sh*VdcC_21R | GGTGGTGGTGCTCGATTAGCGGTTCTGGTTTTTCAG |
| *Sh*VdcC_30R | GTGGTGGTGCTCGAGGCGGTTCTGGTTTTTCAG |
| *Sh*VdcD_28F | CGCGCGGCAGCCATATGAAATGTCATCGTTGTGG |
| *Sh*VdcD_21F | AAGGAGATATACATAATGAAATGTCATCGTTGTGG |
| *Sh*VdcD_21/28R | GGTGGTGGTGCTCGATCATTTTTTCAGCGGAGG |
| *Bs*VdcC_21/30F | AAGGAGATATACATATGGCATACCAAGATTTTCGTGAATTTC |
| *Bs*VdcC_21R | GGTGGTGGTGCTCGAGTTATTATTCATCAGATCCATCAGTTTTTGTTC |
| *Bs*VdcC_30R | GGTGGTGGTGCTCGAGTTCATCAGATCCATCAGTTTTTGTTC |
| *Bs*VdcD_28F | CGCGCGGCAGCCATATGCATACCTGCCCGCG |
| *Bs*VdcD_21F | AAGGAGATATACATATGCATACCTGCCCGCG |
| *Bs*VdcD_21/28R | GGTGGTGGTGCTCGAGTTATTACGCTTTGCGTTCCGGAAC |
| *Sh*VdcD-V59X_Q5F | CCAACATGTAGGTTATTCCGC |
| *Sh*VdcD-V59X_Q5R | CGATTTTGTTATCGTCCAGTTTG |
| *Sh*VdcC-L182V_Q5F | TATTCAGGCAGTGGCAATGCATG |
| *Sh*VdcC-L182V_Q5F | CCAACACGATCACGATCTTTAAC |

**Supplementary Table 2: Primers used in this study**. Primers were used in pairs denoted by gene product and vector number.

| Name | C Subunit | D Subunit | UbiX |
| --- | --- | --- | --- |
| *Sh*VdcCX | pET30a |  | pCDFDuet |
| *Sh*VdcDX |  | pET28a | pCDFDuet |
| *Sh*VdcC | pET30a |  |  |
| *Sh*VdcD |  | pET28a |  |
| *Sh*VdcCDX | pET21a | pET28a | pCDFDuet |
| *Sh*VdcCD^N^* | pET21a | pET28a |  |
| *Sh*VdcC^C^D* | pET30a | pET21a |  |
| *Sh*VdcCD^V59X^ | pET21a | pET28a |  |
| *Bs*VdcCX | pET30a |  | pCDFDuet |
| *Bs*VdcDX |  | pET28a | pCDFDuet |
| *Bs*VdcC | pET30a |  |  |
| *Bs*VdcD |  | pET28a |  |
| *Bs*VdcCDX | pET21a | pET28a | pCDFDuet |

**Supplementary table 3: BL21 (DE3) transformants produced.** Coexpression of proteins was achieved by transforming competent cells with multiple plasmids.

*two transformants were made for *Sh*VdcCD expression, where the tags were on either subunit of the complex. *Sh*VdcCD using N-terminally tagged *Sh*VdcD in pET28a was the preferred construct.

**Supplementary references**

1 Marshall, S. A. *et al.* Oxidative maturation and Structural Characterization of Prenylated-FMN binding by UbiD, a Decarboxylase Involved in Bacterial Ubiquinone Biosynthesis. *Journal of Biological Chemistry*, jbc. M116. 762732 (2017).

2 Leppik, R. A., Young, I. G. & Gibson, F. Membrane-associated reactions in ubiquinone biosynthesis in Escherichia coli. 3-Octaprenyl-4-hydroxybenzoate carboxy-lyase. *Biochimica et biophysica acta* **436**, 800-810 (1976).

3 Aussel, L. *et al.* Biosynthesis and physiology of coenzyme Q in bacteria. *Biochimica et biophysica acta* **1837**, 1004-1011, doi:10.1016/j.bbabio.2014.01.015 (2014).

4 Payne, K. A. *et al.* New cofactor supports alpha,beta-unsaturated acid decarboxylation via 1,3-dipolar cycloaddition. *Nature* **522**, 497-501, doi:10.1038/nature14560 (2015).

5 Aleku, G. A. *et al.* Terminal Alkenes from Acrylic Acid Derivatives via Non‐Oxidative Enzymatic Decarboxylation by Ferulic Acid Decarboxylases. *ChemCatChem* **10**, 3736 (2018).

6 Nagy, E. Z. A. *et al.* Exploring the substrate scope of ferulic acid decarboxylase (FDC1) from Saccharomyces cerevisiae. *Scientific reports* **9**, 1-10 (2019).

7 Payer, S. E. *et al.* Regioselective para-Carboxylation of Catechols with a Prenylated Flavin Dependent Decarboxylase. *Angewandte Chemie International Edition* **56**, 13893-13897, doi:10.1002/anie.201708091 (2017).

8 He, Z. & Wiegel, J. Purification and characterization of an oxygen-sensitive, reversible 3, 4-dihydroxybenzoate decarboxylase from Clostridium hydroxybenzoicum. *Journal of bacteriology* **178**, 3539-3543 (1996).

9 Payne, K. A. P. *et al.* Enzymatic Carboxylation of 2-Furoic Acid Yields 2,5-Furandicarboxylic Acid (FDCA). *ACS catalysis* **9**, 2854-2865, doi:10.1021/acscatal.8b04862 (2019).

10 Payne, K. A. *et al.* Structure and Mechanism of Pseudomonas aeruginosa PA0254/HudA, a prFMN-Dependent Pyrrole-2-carboxylic Acid Decarboxylase Linked to Virulence. *ACS catalysis* **11**, 2865-2878 (2021).

11 Annaval, T. *et al.* Biochemical and structural characterization of TtnD, a prenylated FMN-dependent decarboxylase from the tautomycetin biosynthetic pathway. *ACS chemical biology* **13**, 2728-2738 (2018).
