# Supplementary material for: UbiD domain dynamics underpins aromatic decarboxylation
Source: Nat Commun. 2021 Aug 20;12:5065. doi: 10.1038/s41467-021-25278-z (PMC8379154; doi:10.1038/s41467-021-25278-z)
Supplement: Supplementary file 2 — Description of Additional Supplementary Files [file 41467_2021_25278_MOESM2_ESM.docx]

Description of Additional Supplementary Files

Title: Supplementary Movie 1

Description: Overall domain motion from open to closed (and reversed) for ShVdcCD hexamer.

Title: Supplementary Movie 2

Description: ElNémo analysis of low frequency normal mode of ShVdcCD complex. Domain motion resembles motion seen between open and closed states of ShVdcCD crystal structures.

Title: Supplementary Movie 3

Description: Proposed motions in catalytic cycle of VdcCD, starting from substrate binding.
