# Supplementary material for: UbiD domain dynamics underpins aromatic decarboxylation
Source: Nat Commun. 2021 Aug 20;12:5065. doi: 10.1038/s41467-021-25278-z (PMC8379154; doi:10.1038/s41467-021-25278-z)
Supplement: Supplementary file 6 — Reporting Summary [file 41467_2021_25278_MOESM6_ESM.pdf]

nature portfolio

Corresponding author(s): Stephen A. Marshall
David Leys

Last updated by author(s): Jun 8, 2021

Reporting Summary

Nature Portfolio wishes to improve the reproducibility of the work that we publish. This form provides structure for consistency and transparency in reporting. For further information on Nature Portfolio policies, see our Editorial Policies and the Editorial Policy Checklist.

Statistics

For all statistical analyses, confirm that the following items are present in the figure legend, table legend, main text, or Methods section.

n/a	Confirmed	
☐	☒	The exact sample size (n) for each experimental group/condition, given as a discrete number and unit of measurement
☐	☒	A statement on whether measurements were taken from distinct samples or whether the same sample was measured repeatedly
☒	☐	The statistical test(s) used AND whether they are one- or two-sided<br>*Only common tests should be described solely by name; describe more complex techniques in the Methods section.*
☒	☐	A description of all covariates tested
☒	☐	A description of any assumptions or corrections, such as tests of normality and adjustment for multiple comparisons
☐	☒	A full description of the statistical parameters including central tendency (e.g. means) or other basic estimates (e.g. regression coefficient) AND variation (e.g. standard deviation) or associated estimates of uncertainty (e.g. confidence intervals)
☒	☐	For null hypothesis testing, the test statistic (e.g. F, t, r) with confidence intervals, effect sizes, degrees of freedom and P value noted<br>*Give P values as exact values whenever suitable.*
☒	☐	For Bayesian analysis, information on the choice of priors and Markov chain Monte Carlo settings
☒	☐	For hierarchical and complex designs, identification of the appropriate level for tests and full reporting of outcomes
☒	☐	Estimates of effect sizes (e.g. Cohen's d, Pearson's r), indicating how they were calculated

Our web collection on statistics for biologists contains articles on many of the points above.

Software and code

Policy information about availability of computer code

Data collection	Crystal diffraction data was collected at Diamond using GDA.<br>UV-Vis data was collected using Agilent Cary Win UV software. Kinetics program v. 5.0.0.999<br>HPLC data was collected using Agilent Chemstation (B.04.03)
Data analysis	Prism 8 was used for the generation of graphs, and the determination of means and standard errors.<br>Crystal data was processed with the Phenix suite (1.17.1-3660), coot 0.8.2 and UCSF Chimera 1.13.1. Docking was performed with Rosetta ligand docking (on the ROSIE web server) and AutoDock Vina. Molsoft ICM Pro (version 3.9-2b) also used for docking<br>DynDom web server was used to analyze domain motions.<br>elNémo web server was used for low frequency normal mode analysis

For manuscripts utilizing custom algorithms or software that are central to the research but not yet described in published literature, software must be made available to editors and reviewers. We strongly encourage code deposition in a community repository (e.g. GitHub). See the Nature Portfolio guidelines for submitting code & software for further information.

Data

Policy information about availability of data

All manuscripts must include a data availability statement. This statement should provide the following information, where applicable:

- Accession codes, unique identifiers, or web links for publicly available datasets
- A description of any restrictions on data availability
- For clinical datasets or third party data, please ensure that the statement adheres to our policy

The atomic coordinates and structures factors (codes 7AE4, 7AE5 and 7AE7) have been deposited to the Protein Data Bank (http://www.pdb.org). Other data will be

7AE4 (doi.org/10.2210/pdb7AE4/pdb), 7AE5 (doi.org/10.2210/pdb7AE5/pdb), 7AE7 (doi.org/10.2210/pdb7AE7/pdb)

made available on request to the corresponding authors. Modelling data uploaded to Zenodo (10.5281/zenodo.5101186), source data file provided in upload.

Field-specific reporting

Please select the one below that is the best fit for your research. If you are not sure, read the appropriate sections before making your selection.

- [x] Life sciences
- [ ] Behavioural & social sciences
- [ ] Ecological, evolutionary & environmental sciences

For a reference copy of the document with all sections, see nature.com/documents/nr-reporting-summary-flat.pdf

Life sciences study design

All studies must disclose on these points even when the disclosure is negative.

Sample size	No calculations were performed to determine sample size. Activity data were taken in duplicate or triplicate as is standard for assays of this type. Sample sizes were decided based on common practice in the field. (e.g crystal data collection to obtain >95% completeness) Activity assays performed in duplicate/triplicate (https://doi.org/10.1038/s42004-020-00368-z)
Data exclusions	No excluded data
Replication	Data were replicated Data presented from technical replicates. Independent replication attempts successfully showed trend.
Randomization	N/A No randomization necessary, this is not clinical study. Experiments did not need allocation to groups.
Blinding	N/A No blinding necessary, this is not clinical study. Groups not allocated.

Reporting for specific materials, systems and methods

We require information from authors about some types of materials, experimental systems and methods used in many studies. Here, indicate whether each material, system or method listed is relevant to your study. If you are not sure if a list item applies to your research, read the appropriate section before selecting a response.

Materials & experimental systems

n/a	Involved in the study	
x		Antibodies
x		Eukaryotic cell lines
x		Palaeontology and archaeology
x		Animals and other organisms
x		Human research participants
x		Clinical data
x		Dual use research of concern

Methods

n/a	Involved in the study	
x		ChIP-seq
x		Flow cytometry
x		MRI-based neuroimaging
